# Organ Donation in Marfan Syndrome: Is It a Case to Stretch Boundaries?

**DOI:** 10.7759/cureus.83913

**Published:** 2025-05-11

**Authors:** Sandeep A Jayasekera, Suchintha B Tillakaratne, Gayana Mahendra, Rohan C Siriwardana

**Affiliations:** 1 Surgery, Colombo North Centre for Liver Diseases, Faculty of Medicine, University of Kelaniya, Colombo, LKA; 2 Gastrointestinal Surgery, Colombo North Centre for Liver Diseases, Faculty of Medicine, University of Kelaniya, Colombo, LKA; 3 Histopathology, Colombo North Centre for Liver Diseases, Faculty of Medicine, University of Kelaniya, Colombo, LKA

**Keywords:** connective tissue disorder, liver transplantation, marfan syndrome, organ donation, vascular fragility

## Abstract

Marfan syndrome is an autosomal dominant connective tissue disorder caused by mutations in the *FBN1* gene. It primarily affects the cardiovascular, ocular, and skeletal systems. Despite improved survival outcomes, organ donation from these patients remains rare due to concerns of vascular fragility. A 44-year-old male with Marfan syndrome was considered for organ donation following brain stem death due to a massive intracerebral hemorrhage caused by a ruptured arteriovenous malformation. He had a history of mitral valve replacement and kyphoscoliosis. His liver and renal function tests were within normal limits. Consent for donation was subsequently obtained. Intra-operative findings revealed a grossly deformed liver and thin, friable vasculature, prompting the retrieval team to abandon the procedure. Biopsies from the liver and iliac artery were obtained for histopathological evaluation. Liver histology showed mild fibrosis without significant steatosis or inflammation. Iliac artery biopsy revealed cystic medionecrosis (with risk of aneurysmal formation), typically seen in Marfan syndrome. Although limited literature exists on organ donation in Marfan syndrome, rare successful liver transplants have been reported. However, vascular fragility and risk of aneurysmal changes remain a concern. Our intra-operative findings and supporting histology reinforce the challenges in safely retrieving organs from donors with Marfan syndrome. While expanding donor criteria is crucial in meeting growing transplant demands, this case highlights the importance of careful intra-operative assessment and maintenance of a low threshold for abandonment in the presence of concerning vascular or organ abnormalities in donors with Marfan syndrome.

## Introduction

Marfan syndrome is an autosomal dominant connective tissue disorder, characterized by involvement of the cardiovascular, ocular, and skeletal systems. Mutations in the *FBN1* gene encoding fibrillin-1 are responsible for the disease [1]. Although modern medicine has improved the survival of these patients, implications for organ donation remain marginal due to the multi-systemic nature of the condition. The feasibility of solid organ transplantation, especially the liver, remains poorly understood [2]. This is one of the few documented intra-operative assessments of liver suitability in a deceased donor with Marfan syndrome.

## Case presentation

A 44-year-old male with Marfan syndrome and a BMI of 18 kg/m^2^, who had a history of mitral valve replacement for mitral valve prolapse, had a sudden onset of loss of consciousness and collapse. A CT scan of his brain showed a massive left-sided intracerebral hemorrhage. The cause was suspected to be a ruptured cerebral arteriovenous malformation. The patient never regained consciousness, and it was concluded that he had brain stem death two days after the index event. He was considered for organ donation due to his young age and absence of any other significant medical comorbidities.

His family consented to the donation, and he was subsequently worked up for it. His liver function tests were normal, as were his renal functions. However, marked distortion of the body was noted, caused by deformities due to significant kyphoscoliosis. A multidisciplinary meeting was conducted amongst the organ retrieval team, and the decision was made to retrieve his organs.

**Figure 1:**
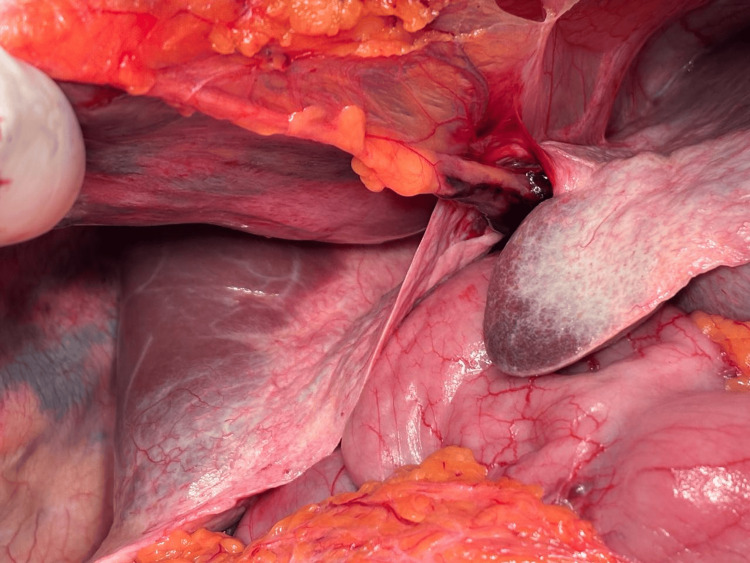
Intra-operative view of the liver of the donor with Marfan syndrome showing abnormal macroscopic morphology.

Upon opening of the abdomen, it was noted that the macroscopic morphology of the liver was grossly deformed due to kyphoscoliosis (Figure 1). However, there was no macroscopic evidence of cirrhosis. The kidneys appeared structurally normal but exhibited thin, friable vasculature. After an intra-operative discussion between the two consultant surgeons performing the retrieval, it was decided not to go ahead, and instead, a liver biopsy and iliac artery biopsies were taken to better understand the underlying pathology.

The liver biopsy demonstrated mild fibrosis (grade I-II) without significant steatosis or cholestasis. Occasional collections of lymphoid cells were seen. Lobular inflammation and interface hepatitis were minimal, without any evidence of malignancy.

**Figure 2:**
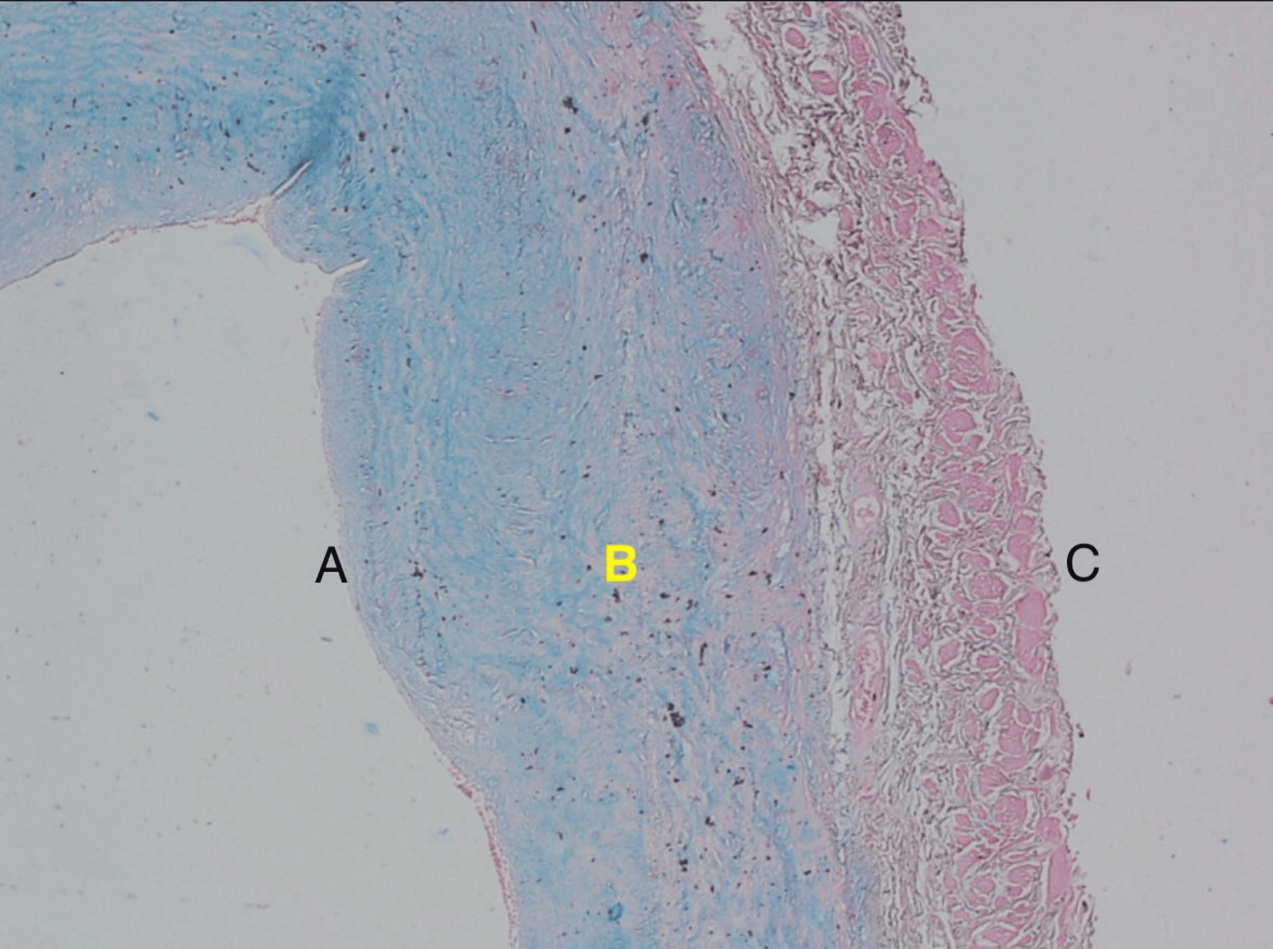
Histological section of an iliac artery from the potential donor with suspected Marfan syndrome, stained with Masson's trichrome (original magnification, x100). The tunica media demonstrates features consistent with cystic medionecrosis. A: Tunica intima. B: Tunica media demonstrating cystic medionecrosis. C: Tunica adventitia.

The iliac artery biopsy showed a muscular artery with largely disrupted internal and external elastic laminae. Prominent myxoid degeneration and cystic medionecrosis of the wall were noted, with Alcian blue-positive mucin deposition. Vessel wall inflammation was minimal, and dissections or aneurysms were not seen. These histological changes were typical of those commonly seen in the vasculature of Marfan syndrome patients (Figure 2).

## Discussion

The majority of donors available for deceased donor liver transplant (DDLT) are not ideal, especially in the current day and age with the ever-increasing incidence of metabolic syndrome [3]. All donors should be considered, including those with congenital disorders that may theoretically impair organ integrity. In the case of Marfan syndrome, the relatively high incidence of vascular complications compared to the normal population poses a challenge when it comes to a successful donation [4].

There is a clear paucity of data on organ retrieval from Marfan syndrome patients, especially concerning the liver. Only a few successful cases have been documented. A Japanese group reported a DDLT done from a Marfan syndrome donor, having a one-year survival without complications [5]. In that case, macroscopically, the liver looked healthy with iliac arteries having normal elasticity on intraoperative palpation. In the very limited literature available, there are no reports of recipient complications following liver transplant (LT) where the donor had Marfan syndrome.

The documented incidence of visceral artery changes in Marfan syndrome is 0.4-2% [6]. Two hepatic artery complications have been documented previously by Santiago-Delpin et al. [7] in a non-transplant setting. In this patient, the donor at the age of 44 years showed significant microscopic changes involving the elastic lamina. The healing process and the endurance of the anastomosis and the graft vessels to the flow turbulence with these microscopic changes are highly unpredictable. Hence, accepting a graft with a Marfan syndrome background needs reconsideration.

In the case described in this report, the liver was grossly abnormal, with the iliac arteries feeling brittle and unhealthy on intraoperative palpation as well. Concerns regarding vascular fragility prevented the safe retrieval of the organs for transplantation.

## Conclusions

The ever-increasing need for organ donation in the current clinical context of the world demands consideration of donors previously disregarded due to congenital anomalies. However, retrieval teams should maintain a low threshold for abandonment in donors with Marfan syndrome due to the unpredictability of microscopic vascular changes.
